# The implausibility of ‘usual care’ in an open system: sedation and weaning practices in Paediatric Intensive Care Units (PICUs) in the United Kingdom (UK)

**DOI:** 10.1186/s13063-015-0846-3

**Published:** 2015-07-31

**Authors:** Bronagh Blackwood, Lyvonne Tume

**Affiliations:** School of Medicine, Dentistry and Biomedical Sciences, Queen’s University Belfast, University Road, Belfast, BT7 1NN Northern Ireland UK; School of Health, PICU, Alder Hey Hospital, Eaton Road, Liverpool, Merseyside L12 2AP UK; University of Central Lancashire, Fylde Road, Preston, Lancashire PR1 2HE UK

**Keywords:** Sedation, Ventilator weaning, Intensive care, Paediatrics, Implementation, Trials

## Abstract

**Background:**

The power of the randomised controlled trial depends upon its capacity to operate in a closed system whereby the intervention is the only causal force acting upon the experimental group and absent in the control group, permitting a valid assessment of intervention efficacy. Conversely, clinical arenas are open systems where factors relating to context, resources, interpretation and actions of individuals will affect implementation and effectiveness of interventions. Consequently, the comparator (usual care) can be difficult to define and variable in multi-centre trials. Hence outcomes cannot be understood without considering usual care and factors that may affect implementation and impact on the intervention.

**Methods:**

Using a fieldwork approach, we describe PICU context, ‘usual’ practice in sedation and weaning from mechanical ventilation, and factors affecting implementation prior to designing a trial involving a sedation and ventilation weaning intervention. We collected data from 23 UK PICUs between June and November 2014 using observation, individual and multi-disciplinary group interviews with staff.

**Results:**

Pain and sedation practices were broadly similar in terms of drug usage and assessment tools. Sedation protocols linking assessment to appropriate titration of sedatives and sedation holds were rarely used (9 % and 4 % of PICUs respectively). Ventilator weaning was primarily a medical-led process with 39 % of PICUs engaging senior nurses in the process: weaning protocols were rarely used (9 % of PICUs). Weaning methods were variably based on clinician preference. No formal criteria or use of spontaneous breathing trials were used to test weaning readiness. Seventeen PICUs (74 %) had prior engagement in multi-centre trials, but limited research nurse availability. Barriers to previous trial implementation were intervention complexity, lack of belief in the evidence and inadequate training. Facilitating factors were senior staff buy-in and dedicated research nurse provision.

**Conclusions:**

We examined and identified contextual and organisational factors that may impact on the implementation of our intervention. We found usual practice relating to sedation, analgesia and ventilator weaning broadly similar, yet distinctively different from our proposed intervention, providing assurance in our ability to evaluate intervention effects. The data will enable us to develop an implementation plan; considering these factors we can more fully understand their impact on study outcomes.

## Background

Many trials in intensive care require a pragmatic design. In contrast to explanatory trials, performed to test whether an intervention works under optimal situations akin to a closed system, pragmatic trials evaluate the effectiveness of interventions in real-life current practice conditions that are influenced by various factors operating in open systems [1]. Because pragmatic trials are performed to determine whether the intervention can improve current practice, they often have a ‘usual care’ control group. The care received by this control group is supposed to reflect the care as usually received by patients in daily practice. The Declaration of Helsinki states that the “benefits, risks, burdens, and effectiveness of a new method should be tested against those of the best current prophylactic, diagnostic, and therapeutic methods” [2] and it is upon this principle that the randomised controlled trial is performed. However, in many cases the ‘best current method’ has not yet been identified or validated and ‘usual care’ may include wide variation in practice styles. The term ‘usual care’ then becomes problematic because it implies a uniform practice standard, so what may be considered ‘usual’ in one context, may be ‘unusual’ in another. Even in cases where good evidence is available to guide clinicians, there may still be substantial variability and inconsistency in usual care [3]. It is the inconsistency in practice that makes ‘usual care’ difficult to understand and describe and limits its value in the control group of a clinical trial. It follows that if we want to compare a new intervention (such as a clinical pathway or protocolised care) against usual care, it is important to understand what constitutes ‘usual care’ in an open system such as we describe, in order to determine if, and how, it differs from the new intervention.

Despite the importance of optimising sedation and minimising time on mechanical ventilation, there is limited guidance in the paediatric literature to direct practice on reducing sedation and discontinuing ventilation. Consensus guidelines on sedation developed in the United Kingdom (UK) and Germany [4, 5] recommend regular assessment using validated tools and titration of sedatives to desired levels. However, a systematic review of studies evaluating sedation practice in paediatric intensive care reported that sedation is often suboptimal and seldom systematically evaluated [6]. Nevertheless, even when a patient’s sedation levels are evaluated and linked to a sedation protocol to guide sedative infusion rates, adequate sedation is not guaranteed. A recent UK multi-centre trial (n = 10 sites; n = 129 participants) of sedatives in paediatric intensive care units (PICUs), employing frequent use of a sedation scoring tool for assessing sedation, reported that 25 % of patients were adequately sedated for only 58 % of the time and they were more likely to be over-sedated than under-sedated [7].

With regards discontinuing mechanical ventilation, there is no standard method of weaning. A review of weaning and extubation reported that most paediatric studies either described weaning practice in single PICUs or retrospectively sought to identify predictors of successful weaning [8]. Prospective studies of paediatric ventilator weaning practice are scarce. While a meta-analysis of 17 trials in adults revealed that protocol-based weaning reduces duration of mechanical ventilation and ICU stay [9], the evidence is limited in children [10]. Applying similar methods used in adult weaning, the largest trial in 294 children [11] demonstrated that a daily evaluation to check readiness for weaning combined with a spontaneous breathing test reduced total duration of mechanical ventilation by 32 hours (95 % confidence interval (CI) 8 to 56; *P* = 0.01). Two small pilot studies using computer-based weaning protocols also reported reductions in duration of total ventilation, albeit non-significant [12, 13]. There is growing interest in using weaning protocols to guide discontinuation of ventilation [14], but as a result of the paucity of evidence, the adoption of weaning protocols and nurse engagement in the weaning process in the UK is much lower in PICUs (18 %) than adult ICUs (54 %) [15]. Existing evidence supports a realistic expectation that weaning protocols will reduce the duration of mechanical ventilation, but an appropriately powered trial is required to provide a reliable estimate of effects against usual care.

Trials of sedation and ventilator weaning are complex because they involve a number of components that act inter-dependently. These include patient monitoring and assessment, measurement and evaluation of sedation and ventilation requirements, practitioner education and training, and inter-professional collaboration. The multi-component structure of such an intervention can challenge evaluation because it needs to provide information on whether the intervention was delivered as intended [16]. It follows, that to understand the impact of a new clinical intervention on sedation and ventilator weaning, one first requires an understanding of how the processes currently operate. An additional consideration is that trials in the PICU population often require multi-centre involvement in order to recruit an adequate sample size. The intervention may be implemented and received in different ways across different PICUs and the implementation process can be affected by variables related to organisational structure, functioning and research capacity (i.e. resources, training), thus the variance between PICUs needs to be understood [17]. Successful implementation of research evidence or clinical interventions is also dependent upon having robust believable evidence, a receptive context, and appropriate facilitation [18]. Thus important groundwork exploring these areas is essential in order to plan an implementation strategy and other investigators of complex interventions (such as delirium in the ICU) are adopting similar strategies [19].

There is extensive and convincing evidence confirming the strong impact of careful implementation on outcomes [17]. A review by Durlak and DuPre of the relationship between implementation and outcomes highlighted that mean effect sizes are two to three times higher when interventions are carefully implemented and free from serious problems [17]. From the studies included in their review they developed a framework comprising factors that affected the implementation process. We felt it important to know and understand these factors to provide essential baseline information prior to submitting a grant application to evaluate protocolised care in sedation and ventilation weaning in UK PICUs. Consequently, using their framework, we set out to identify what constituted ‘usual care’ practices and to ascertain factors that may impact on the implementation process in sites registering interest in participating in the trial.

## Methods

### Design

We used a fieldwork approach to gather baseline information framed around the fieldwork process outlined by the National Institute of Health and Care Excellence [20]. To undertake this work, we obtained an ‘Enabling Research Award’ from the Health and Social Care, Public Health Agency, Research and Development (PHA R&D) Division, Northern Ireland, UK, which supports applications to the UK National Institute of Health Research (NIHR). Our fieldwork methods involved observation, individual and multi-disciplinary group discussions with PICU staff in 23/29 PICUs in England, Wales and Northern Ireland visited between June and November 2014. Following the visits, we held a collaboration meeting (November 2014) to discuss the proposed intervention and trial design, and how best implementation could be facilitated in a multi-centre trial. Meeting participants included a doctor and nurse from 20 potential sites, and the research team of clinical trialists, methodologists, statisticians and health economists.

### Consent

Discussions with PHA R&D Division and the University’s Research Governance and Integrity Office established that the fieldwork was not considered to be research, therefore an ethical review or additional governance approvals were not required.

### PICU engagement

A trial outline was presented at a Paediatric Intensive Care Society Specialist Group (PICS SG) in September 2013 and the topic was ranked as the highest priority in the prioritisation survey of PICS members. We subsequently presented the trial proposal at a PICS SG meeting in June 2014. We approached PICU consultant intensivists attending the meeting, and emailed those not attending to request permission to visit their units to discuss the trial with key multi-professional staff: we followed this up with an email to confirm.

### Data collection

Fieldwork was conducted mainly by BB and LT together and on four occasions by LT alone. Our aim was to obtain relevant information on factors that may affect the implementation process. These factors were derived from the work of Durlak and DuPre [17], adapted for the PICU visits and are presented in Table 1. We used a fieldwork data collection sheet to ensure we captured consistent information from each PICU relating to these factors (Appendix). Each site visit began with a presentation of the trial to ICU staff followed by an open forum discussion to facilitate understanding and ascertain interest. We undertook focused discussions with key medical, nursing, allied health professional and research staff to collect information about usual care practice in sedation and ventilator weaning and other factors affecting the implementation process. We were shown around the PICU, which provided further opportunity to collect relevant information (such as pain, sedation and weaning guides) and view usual recording practices. BB and LT wrote independent notes derived from the discussion with staff and observations on separate data collection forms and debriefed after each visit to collate information, which was captured onto one data collection sheet. Where necessary, we followed up with site staff to confirm queries or missing information.

**Table 1:** Factors affecting implementation

PICU organisational characteristics	Unit size and staffing	Number of beds; staff ratios; skill mix; educational provision; night-time medical staffing
PICU staff characteristics	Perceived need for intervention	Extent to which the proposed intervention is relevant to local needs
	Perceived benefits of intervention	Extent to which the intervention will achieve benefits desired at the local level
	Self-efficacy	Extent to which staff feel they will be able to do what is expected
	Skill proficiency	Possession of the skills necessary for implementation
Characteristics of the intervention	Compatibility	Extent to which the intervention fits with the PICU’s priorities and needs.
Organisational capacity	Positive work climate	Staff views about trust, collegiality, and methods of resolving disagreements
	Organisational norms regarding change	Engagement in research and previous trials, openness to change
	Integration of new intervention	Extent to which the unit can incorporate the intervention into its existing practices and routines
	Shared vision	Consensus, commitment, staff buy-in
Usual care processes	Pain and sedation management	Pain, sedation and withdrawal assessment tools in current use, frequency of assessment and compliance. Sedation protocols, sedatives used, nurses’ role in sedation titration. Use of sedation and neuromuscular blockade holidays
	Ventilator weaning practice	Weaning protocols, types of weaning, usual methods employed, nurses’ engagement in weaning process
Other practices and processes	Shared decision-making	The extent to which staff collaborate in determining what will be implemented and how
	Communication	Effective mechanisms encouraging frequent and open communication
	Formulation of tasks	Procedures that enhance planning and contain clear roles and responsibilities relative to implementation
Specific staffing considerations	Leadership	Extent to which senior staff clearly support and encourage providers during implementation
	Programme champion	An individual who is trusted and respected by staff and administrators, and who can rally and maintain support for the innovation, and negotiate solutions to problems that develop
Intervention support system	Training	Approaches to ensure staff proficiencies in the skills necessary to conduct the intervention
	Research assistance	Resources available once implementation begins, research nurse availability, provision for training, training of new staff, and mechanisms to promote local problem-solving efforts
	Prior experience	Barriers and facilitators to previous trial implementation

*PICU* Paediatric Intensive Care Unit

### Analysis

Quantitative data relating to staff numbers and unit beds were collated in an Excel database and summarised using descriptive statistics (proportions, range, median [interquartile range, IQR]). Narrative and observational textual data relating to each of the factors outlined in the data collection sheet were entered into tables in Word for ease of documenting. These factors were categorised and summarised for reporting.

## Results

### PICU organisational characteristics

The 23 PICUs ranged in size from 5 to 31 (median 12, IQR 8, 18) funded beds for intensive care patients. All hospitals visited had high-dependency beds ranging from 2 to 15 (median 4, IQR 3, 9) that could be used as intensive care beds if necessary: three hospitals had separate high-dependency units. Each PICU had a 1:1 registered nurse to patient ratio for invasively ventilated children. The consultant to patient ratio varied depending on time of day and the size of the unit, from 1:23 (overnight) in larger units to 1:5 in the day for smaller units. Consultant medical staff were always resident 24 hours a day/7 days a week in three larger units, otherwise they were present during daytime and early evening hours and covered on-call from home at night. Clinical patient information was recorded by electronic clinical information systems exporting physiological data directly to electronic health records in eight (35 %) PICUs; the others used paper-based recording charts. In 11 (48 %) PICUs, medical staff conducted morning handover in a conference room before undertaking a walk-around ward round.

### Usual care processes

#### Pain and sedation management

Analgesia and sedation medication types varied according to the child’s age and condition. All units used intravenous morphine as the primary opiate with 20 PICUs (87 %) using morphine in combination with midazolam for sedation. In six units (6/23, 26 %), clonidine was administered either as the main sedative drug or for children younger than 3–6 months. Clonidine was administered intravenously in all but one unit where it was prescribed orally. Use of neuromuscular blockade was generally rare and reserved for cases such as those with ‘difficult and precarious airways’; severe traumatic brain injuries; post-operative cardiac surgical infants with delayed sternal closure; children receiving extracorporeal membrane oxygenation; or children with unstable pulmonary to systemic blood flow.

Pain was not formally scored in three PICUs (13 %). In 20 (87 %) units that scored pain levels, age-appropriate tools were used (CRIES [21], Faces, Legs, Activity, Cry and Consolability (FLACC) [22] and Faces [23], with two units using the COMFORT [24] score (for distress) as a measure of both pain and sedation. Sedation level was scored in 21 PICUS (91 %) and the most common assessment tool was the standard COMFORT score used by 19 PICUs (83 %) measured at hourly or 4-hourly intervals. Two units used invalidated scoring tools to assess sedation. Seven PICUs (30 %) reported having sedation guidelines for titrating sedation; three units reported they did not use them often and two units linked actions in sedation guidelines to the COMFORT score sedation level. Only one PICU practiced daily sedation holds and had done for a number of years. Sedation holds involve temporarily stopping sedative infusions until the child wakens enabling assessment of further sedation requirements. Analgesia and sedative withdrawal symptom scoring was undertaken in 11 PICUs (48 %) and the tools used were the Withdrawal Assessment Tool [25] (n = 5); Sedation Withdrawal Score [26] (n = 4); Sophia Observation withdrawal Symptoms scale [27] (n = 1) or unsure (n = 1). All but one unit stated they attempted to ensure a daily relaxant hold, but were not confident this always happened; five PICUs (22 %) were confident that children always received a daily relaxant hold.

In 22 PICUs (95 %), nurses were authorised to titrate intravenous sedative and analgesic infusions within a medically prescribed infusion range or administer an intravenous bolus according to patient assessment. In one PICU, nurses could not alter the infusion rate without medical staff sanction, but were authorised to administer up to three intravenous boluses. When probed about assessment and subsequent management, nurses reported their decisions to titrate intravenous sedative medication were seldom associated with the scores on the sedation assessment tool and they revealed they were more likely to increase than decrease medication. Furthermore, the trends in daily sedation level scores were rarely discussed in ward rounds and were not considered in formulating sedation plans. Nurse compliance with sedation scoring was reported to be a problem in many units.

#### Ventilator weaning practice

In nine PICUs (39 %), a proportion of senior nurses were engaged in weaning children from mechanical ventilation and this included reducing ventilator support to a pre-extubation level (five PICUs) or to extubation (four PICUs). Weaning was undertaken by nurses following a locally developed weaning protocol (two PICUs), based on senior clinical experience (three PICUs), or following successful completion of an academic weaning ventilation course (four PICUs).

None of the PICUs reported having a formal set of criteria to determine readiness to wean or extubate. Spontaneous breathing trials (SBTs) were conducted infrequently in PICUs: one PICU reported it was common practice to conduct an SBT using either 5 cmH20 continuous positive airway pressure (CPAP) for 30–120 minutes and three PICUs reported it was occasionally practised. Methods of weaning varied across and within PICUs, although three PICUs used a common ‘rules of five′ (or six) for 2 hours to test readiness for extubation referring to CPAP 5 cm H20, positive end-expiratory pressure 5 cm H20 and pressure support 5 cm H20. Only one unit used an automated weaning mode (neurally adjusted ventilatory assist [NAVA®]) although many of the ventilators used could support this application. Weaning to extubation was generally only conducted during the day shift unless there was adequate on-site senior medical cover at night.

### Intervention support systems

Our fieldwork data indicated that each PICU had at least one nurse educator responsible for providing mandatory in-service training courses and new staff induction. On-site training included pain and sedation assessment and generally mechanical ventilation, although specific training in weaning patients from mechanical ventilation was not. Academically accredited weaning courses were delivered on-site in four PICUs within two hospital sites and attended by a small proportion of senior nurses depending on local PICU needs. The majority of sites used the COMFORT [24] sedation assessment tool because it was required in the ‘Safety profiLe, Efficacy and Equivalence in Paediatric intensive care Sedation’ (SLEEPS) multi-centre trial of midazolam and clonidine, of which eight were participating sites [7]. PICU educators had experience of rolling out this type of training for trials. They emphasised that it created intensive effort and increased workload in a tight timeframe, but that it was manageable with additional support, usually provided by research nurses attached to the trial.

Seventeen PICUs (17/23 74 %) had been involved in large multi-centre interventional paediatric trials (Control of Hyperglycaemia in Paediatric intensive care (CHIP) [28], Safety profiLe, Efficacy and Equivalence in Paediatric intensive care Sedation (SLEEPS) [7] , CATatheter infections in CHildren (CATCH) [29], Therapeutic Hypothermia After Pediatric Cardiac Arrest (THAPCA) [30]. One PICU had a dedicated research nurse funded by the hospital. In 19 (83 %) PICUs, funding to undertake screening, recruitment, consent and data collection by research nurses or data managers was obtained either from specific trial funding or from the National Institute of Health Research, Clinical Research Network. The barriers to previous trial implementation were identified as the complexity of the intervention; lack of belief in the benefit of the intervention; and inadequate training. Facilitating factors were senior staff buy-in and research nurse provision. Effective leadership and having a dedicated research nurse who could help orchestrate implementation and training were seen as crucial.

### Other factors affecting implementation

It was important to explore the staff’s perceptions and belief in the need for the sedation and ventilator weaning protocol, because implementation will be enhanced if staff are committed to the study, feel confident in their ability to undertake the intervention and possess the essential skills. Fieldwork interviews revealed that all units visited were receptive to the intervention and expressed keenness to participate in the trial; this came across most strongly from nursing staff. Those units unable to provide an assurance to participate did so because of their organisational capacity and engagement in other trials (n = 2)

### Collaboration meeting

A collaboration meeting was held on 25 November 2014 and was attended by 42 participants. These included nurses and doctors from 20 PICUs who had expressed an interest in participating in the trial; and the research team including clinical trialists, methodologists, statisticians and health economists. Summarised feedback on usual care practices from the visited PICUs was presented at the collaboration meeting. Subsequent discussions ensued about the design of the trial and its impact on PICUs, the implementation strategy and the intervention itself. The aims of the meeting were to discuss incongruities, and encourage a shared vision and buy-in from sites. We collected further information on usual care practices pertaining to sedation and ventilator weaning and through discussion established a compromise on an evidence-based intervention that would be agreeable to participating sites.

### The proposed intervention

Broadly speaking, the new intervention will be designed to engage all PICU staff from the junior bedside nurse to the medical consultant in sedation and weaning tailored to their level of experience. The intervention protocol will include monitoring and assessing sedation levels and titrating intravenous infusions to achieve optimal sedation within a prescribed range, daily assessment of the child’s readiness for an SBT using an agreed set of clinical criteria, followed by a maximum 2-hour SBT if the criteria are met. Participants agreed that ventilation parameters for the SBT should be considerably lower than those in the readiness criteria in order to more reliably ascertain the child’s ability to tolerate spontaneous breathing and extubation.

## Discussion

This pre-trial exploration of baseline practice is the first in the UK paediatric intensive care population to have reported a rigorous process using an evidence-based framework prior to trial set-up to ascertain usual care and factors that might affect implementation. As a result, it has provided valuable information about ‘usual care’ in the open system of UK PICU practice around sedation and ventilation weaning. The methods we used in gathering this information may also provide useful guidance for future clinical trial design.

We found that pain and sedation practices in UK PICUs are broadly similar in terms of drug usage, pain and sedation assessment tools and timing. Sedation assessment was commonly scheduled hourly to 4-hourly during the day, but scores were rarely linked to actions for drug titration. This resulted in reported low compliance with scheduled assessment times. Relatively few PICUs (30 %) had written sedation guidelines and only one PICU practised sedation holds. This is in contrast to adult intensive care units where sedation withholding is a recommended part of the ‘ventilator care bundle’, as outlined by the UK Department of Health [31], the Institute for Health Care Improvement [32] and recommended by the Surviving Sepsis Campaign [33]. The purported reasons for not introducing sedation holds in children were concerns regarding agitation leading to adverse events and consequential parental discontentment. Consensus guidelines on sedation and analgesia in critically ill children were developed by the UK PICS [4], but it is notable from our fieldwork that some of the recommendations remain unimplemented (Table 2). Furthermore, results from the recent UK SLEEPS trial evaluating midazolam and clonidine [7] reported fewer than half (47 %) of participating PICUs routinely scored for sedation withdrawal syndrome and had no written guidelines to guide sedation/opiate drug weaning. Their findings showed low proportions of children who were adequately sedated >80 % of the time (34.4 %, 30.5 %) and high proportions of patients with withdrawal symptoms (46.7 %, 52.6 %). Given the accumulating evidence about the benefits of sedation protocols and holidays in the adult population [34] and associations with improved outcomes in children [35], prospective investigations in the paediatric population are warranted and are likely to produce benefits for children in UK PICUs. For the purpose of our trial, it will be important to attain full support and engagement from all staff in a sedation protocol linking assessment and sedative titration. This will require not only further update training on use of the sedation scoring tool, but also willingness to include discussions of sedation scoring in the PICU rounds and encouragement of positive attitudes towards lighter sedation.

**Table 2:** Compliance with the UK Paediatric Intensive Care Society consensus guidelines

Recommendation from 2006 consensus guidelines	Compliance within the observed PICUs
Pain assessment should be performed regularly by using a pain scale appropriate for patient age	In 87 % of PICUs pain is formally assessed and scored
The level of sedation should be regularly assessed using a validated sedation assessment score e.g. COMFORT score	83 % of PICUs use a validated tool, but compliance with regular assessment is low. 8 % of PICUs use a protocol to titrate according to sedation score
The desired level of sedation should be identified for each patient and should be regularly reassessed	
Dosage of sedatives should be titrated to produce the desired sedation level	
The use of clinical guidelines for sedation is recommended	30 % of PICUs have sedation guidelines
The potential for opioid and benzodiazepine withdrawal syndrome should be considered after 7 days of continuous therapy. When subsequently discontinued the doses of these drugs may need to be tapered.	48 % of PICUs assess withdrawal syndrome
Whenever it is safe to do so, continuous infusions of neuromuscular blockade should be discontinued at least 24-hourly until spontaneous movement returns	22 % of the units very confident this was done daily, 95 % said they tried to do this daily

*PICU* Paediatric Intensive Care Unit

Weaning from mechanical ventilation is principally practised according to PICU clinician preference with no common consensus in methods among consultant medical staff. Senior nursing staff and nurses with accredited courses are engaged in weaning, but rarely according to any formalised weaning protocol. In addition, they comprised a relatively small proportion of the nursing staff, which affected continuity of ventilator weaning. In general, the PICUs did not use readiness to wean criteria and SBTs as a method for testing readiness for weaning. In contrast, SBT protocols are the most common weaning methods used in adult ICU studies where protocols have been shown to significantly reduce duration of mechanical ventilation in comparison with non-protocolised weaning [9]. SBTs, if used in UK PICUs, are often only performed for a few minutes using an anaesthetic rebreathing bag attached to the endotracheal tube to confirm the anticipated outcome that the child is ready for extubation, rather than a weaning trial. Unlike adult ICU, randomised controlled trials of weaning protocols in paediatric units are rare [10] and studies of SBTs have mainly focused on predicting successful extubation [8, 36, 37]. For the purpose of our trial, it will be necessary for units to encourage assessment of readiness for an SBT on a daily basis using a set of simple-to-measure criteria. Daily assessment, even during the acute phase of illness, will facilitate embedding the assessment into daily routine and promote sustainability in practice. Consideration will be required for training (all staff, particularly junior bedside nurses), incorporating discussion of readiness into the daily ward round, and obligation to undertake an SBT if criteria are met.

With regards trial planning, it is clear the majority of PICUs have limited resources in terms of research nurse capacity. In trials requiring individual patient consent, when research nurse time is funded as part of a trial, there is good participation and recruitment in large interventional multi-centre studies, as evidenced by the CHIP and CATCH trials in particular. Our fieldwork findings suggest that our proposed trial will require a proportion of research nurse support for each participating site.

### Overall completeness and applicability of findings

Our fieldwork was conducted in 23 from a total of 29 National Health Services in England, Wales and Northern Ireland. We purposively did not collect data from the two Scottish children’s hospitals as laws in Scotland governing informed consent preclude their participation in our proposed trial. The data we collected on usual sedation and ventilation weaning practices show broad similarity across the PICUs. We are confident, therefore, that these practices reflect a generalizable trend across hospitals.

### Limitations

Our data were collected from available PICU staff during fieldwork visits and we have not verified the accuracy of these data objectively. However, by meeting a broad range of staff and collecting data in this face-to-face manner, we feel we elicited greater depth of insight than that which could be gained by survey alone. In addition, the visits afforded us the opportunity to obtain copies of relevant guidelines, observations charts and information to corroborate the data.

## Conclusions

We used an evidence-based framework to identify the variance in contextual, organisational and usual care practices that operated within 23 PICUs. This approach was extremely useful in helping us to describe and define some of the ‘openness’ that is so often a feature within pragmatic trials. The benefit of undertaking this baseline work prior to trial commencement is that it has enabled us to plan a robust implementation strategy and consider how identified factors may impact on trial outcomes. We found that usual PICU practices relating to sedation, analgesia and ventilator weaning were broadly similar in England, Wales and Northern Ireland. There was a noticeable lack of sedation and ventilator weaning protocols to guide and support staff in patient assessment and management in these areas. We are confident, having explored factors affecting implementation, that this intervention is compatible with the values and missions of the participating sites. Nevertheless, we are not naïve in understanding the complexities of multi-site research. We intend to extend our evaluation of factors that may impact on implementation by conducting a process evaluation alongside the trial. The process evaluation, informed by the Promoting Action on Research Implementation in Health Services (PARIHS) framework [38], will follow a similar design to that used in a cluster trial in acute care considering the interplay between the intervention, local experience, the PICU context and the implementation process [39]. This will reveal how the sedation and ventilation weaning protocols were received within PICUs, any observed local issues, how the implementation processes performed and the impact on the trial outcomes.

## Appendix

### Data collection sheet

DATA COLLECTION FORM FOR SITE VISITS

Date of visit:

PICU:

Key contact people (with emails):

STAFFING AND UNIT INFRASTRUCTURE

Number of beds ICU high-dependency unit

Number of nursing staff (bodies). Number of medical staff

Number of advanced nurse practitioners, nurse educators or pharmacists?

Number of research nurse/s

PAIN AND SEDATION MANAGEMENT

Does your unit pain score and if so what tool?

Does your unit sedation score and if so what tool?

Does your unit withdrawal score? What tool is used?

Do you set daily goals set formally on withdrawal?

What are your standard sedatives and analgesics used in ventilated children?

Do they differ by patient group?

Do nurses usually alter, increase or decrease sedatives/analgesics? How do they do this?

Do you have sedation guidelines on your PICU? Pain guidelines?

Do you have sedation-targeted flowcharts/algorithms etc. for titrating sedation level?

WEANING SEDATION AND VENTILATION

Do you have weaning sedation guidelines and after how long on sedation/opiates do these get applied?

When do you start withdrawal scoring?

Does your unit use sedation holidays or do any ‘rousable’ tests daily?

Do you undertake a daily extubation readiness test or spontaneous breathing trial? If so how?

Do all nurses sample arterial blood gases on your unit?

What ventilators and ventilation modes do you commonly use?

Do nurses wean ventilation normally on your unit? Is this by protocol or autonomously?

USE OF ALGORITHMS AND IMPLEMENTING THESE ON THE UNIT

Does your unit use other algorithms? Think of one that was successfully implemented, e.g. enteral feeding, goal-directed therapies, etc.

How was it implemented? (What were the barriers/facilitators)

Who are key people on your unit to get things done/brought in?

Been involved in any multi-centre trials before? Which one/s?

Problems with previous trials (implementation, resources, staff buy-in)?

How have previous trials been ‘brought in’? How long did this take?

In-house education/training programme: is it multi-professional or separate – what format?

Any other notes from unit visit:

## Abbreviations

PICU: Paediatric Intensive Care Unit
SBT: spontaneous breathing trial
